# Implementation of dispatcher-assisted cardiopulmonary instructions using the ALERT protocol: preliminary results in Belgium

**DOI:** 10.1186/cc12239

**Published:** 2013-03-19

**Authors:** A Ghuysen, S Stipulante, M El Fassi, A Donneau, V D'Orio, R Tubes

**Affiliations:** 1 CHU - Ulg Liège, Belgium; 2 Liège University, Liège, Belgium

## Introduction

Early bystander cardiopulmonary resuscitation (CPR) is a key factor in improving survival from out-of-hospital cardiac arrest (OOH-CA). The ALERT algorithm, a simple and effective compression-only telephone CPR protocol, has the potential to help bystanders initiate CPR. This study evaluates the effectiveness of the implementation of this protocol in the Liege dispatching centre.

## Methods

We designed a before-and-after study based on a 3-month retrospective assessment of the adult victims of OOH-CA in 2009, before the implementation of the ALERT protocol in the Liege dispatching centre, and the prospective evaluation of the same 3-month period in 2011, immediately after the implementation of this protocol. Data were extracted from ambulance, paramedical and medical intervention teams files, as well as the audio recordings of the dispatching centre.

## Results

There were 233 OOH-CAs detected in the first period and 235 in the second. Victims were predominantly male (59%, both periods), aged 66 and 64 years, respectively. Callers were family members in 52% in 2009 and 64% in 2011. In 2009, only 9.9% victims benefited from bystander CPR, while there were 22.5% in 2011 (*P <*0.0002). Reasons for protocol underuse were: assistance not offered (42.3%), caller remote from the victim (20.6%) or emotionally distressed (15.5%). Mean no-flow time decreased from 253 seconds in 2009 to 168 seconds in 2011 (NS). Ten victims were admitted in ROSC to hospital in 2009 and 13 in 2011 (*P *= 0.09).

## Conclusion

Using the ALERT protocol in the Liege dispatching centre significantly improved the numbers of patients in whom bystander CPR was attempted. Dispatchers must embrace this new opportunity to help callers and be encouraged to accept the responsibility of initiating such assistance.
